# Implementing Prolonged Exposure Therapy in a Community Substance Use Treatment Program: A Qualitative Study

**DOI:** 10.1111/dar.70239

**Published:** 2026-08-26

**Authors:** Steven Curto, Tasha Bulgin, Jordan A. Gette, Margaret Swarbrick, Angela Gonnella, Denise Hien, Sonya B. Norman, Shannon M. Kehle‐Forbes

**Affiliations:** ^1^ Center of Alcohol and Substance Use Studies Rutgers University Piscataway New Jersey USA; ^2^ Graduate School of Applied and Professional Psychology Rutgers University Piscataway New Jersey USA; ^3^ University of Kansas Medical Center Kansas City Kansas USA; ^4^ San Diego School of Medicine University of California La Jolla California USA; ^5^ University of Minnesota Medical School Minneapolis Minnesota USA

**Keywords:** community‐based treatment setting, massed‐prolonged exposure therapy, post‐traumatic stress disorder, prolonged exposure therapy, substance use disorders

## Abstract

**Introduction:**

Post‐traumatic stress disorder (PTSD) commonly co‐occurs with substance use disorders (SUD), yet few community‐based SUD programs incorporate evidence‐based trauma‐focused. Prolonged exposure (PE), including its massed format (M‐PE) with session frequency of 3–4 times per week, is a gold standard intervention for PTSD; however, concerns about client readiness, logistical demands and relapse risk have limited its adoption within SUD settings. This study examined staff perspectives on the feasibility and acceptability of integrating M‐PE into a community‐based SUD program.

**Methods:**

Prior to launching a Hybrid Type 1 effectiveness–implementation trial (Project COMET), we conducted semi‐structured virtual interviews with 15 community clinic staff: providers (*n* = 8), administrators (*n* = 2) and peer specialists (*n* = 5). Interviews were recorded, transcribed and analysed using a rapid qualitative analysis framework with matrix techniques to compare themes across roles.

**Results:**

Four overarching themes captured staff perspectives on integrating M‐PE: (Theme 1) Prior Knowledge and Experiences: Most staff were familiar with EMDR, while direct knowledge of PE/M‐PE was limited. (Theme 2) Perceptions of M‐PE: M‐PE was widely viewed as a promising, structured intervention that fits the pacing and duration of SUD care. (Theme 3) Symptom Reduction and Client Impact: Staff anticipated improvements in PTSD and SUD symptoms through trauma‐focused treatment. (Theme 4) Barriers and Constraints: Participants identified several potential implementation challenges, including logistical barriers and client readiness.

**Discussion and Conclusions:**

Findings suggest that staff generally viewed M‐PE favourably but emphasised the importance of ensuring client readiness and organisational support. Enhancing feasibility and long‐term sustainability may require expanded psychoeducation, targeted provider training and flexible delivery models.

**Trial Registration:** ClinicalTrials.gov identifier: NCT06968832.

## 1 Introduction

### 1.1 Comorbid Post‐Traumatic Stress Disorder and Substance Use Disorder

Substance use disorders (SUD) are prevalent, chronic conditions that pose significant public health challenges [1, 2]. SUD often co‐occurs with post‐traumatic stress disorder (PTSD). Notably, in a nationally representative US sample, 22.3% of individuals with PTSD met criteria for drug use disorders and 46.4% met criteria for any SUD [3]. Individuals with PTSD were 1.5 times more likely to meet criteria for a lifetime SUD and 1.2 times more likely to meet criteria for a lifetime alcohol use disorder than those without PTSD [4]. Among those in SUD residential programs, rates of PTSD increase to 42.5% [5].

Despite the well‐documented comorbidity of SUD and PTSD, treatment systems have typically addressed these conditions separately due to concerns about the well‐being of clients receiving SUD treatment [6, 7]. Providers worry about eliciting negative emotions and potentially increasing drug craving or relapse [6]. There are gaps in knowledge and training regarding the treatment of PTSD + SUD, leading many SUD programs to avoid addressing trauma altogether [8] which impacts access to trauma‐informed care and the underdiagnosis and undertreatment of PTSD [6]. This may contribute to poorer treatment outcomes, including greater psychological distress, lower treatment adherence and responsiveness, increased inpatient hospitalisation and more physical health problems among individuals with co‐occurring SUD and PTSD [9, 10]. However, several evidence‐based therapies have demonstrated effectiveness in treating PTSD+SUD [11, 12].

### 1.2 Evidence Base for Prolonged Exposure and Massed Prolonged Exposure Therapy

Prolonged exposure therapy (PE) is considered one of the most effective treatments for PTSD, with meta‐analyses showing response rates ranging from 65% to 86% [13, 14, 15]. Recent reviews of the PTSD and SUD treatment literature further emphasise that trauma‐focused psychotherapies are among the most effective approaches for individuals with PTSD + SUD [16, 17, 18]. PE uses two exposure methods: (i) in vivo exposures, approaching avoided trauma‐related situations to promote extinction learning and distress tolerance; and (ii) imaginal exposures, revisiting the traumatic memory aloud followed by processing the event to alter clients' inaccurate beliefs [19].

PE is effective among patients with SUD, with studies showing that PTSD symptoms decreased by nearly 50% over treatment and the proportion of clients reporting serious emotional problems also declined with no significant increases in substance use craving [20, 21]. Contrarily, there are high rates of attrition, particularly among those with PTSD + SUD. For example, studies using Concurrent Treatment of PTSD and Substance Use Disorders Using Prolonged Exposure (COPE), an evidence‐based treatment utilising PE and Relapse Prevention [22], found that a substantial proportion of participants discontinued during the 12‐week active treatment phase (i.e., before completing the full course of sessions), with additional attrition observed at the 3‐month follow‐up assessment [23]. Similarly high attrition has been observed in other trauma‐focused and non‐trauma‐focused treatments. For example, in a randomised clinical trial comparing Cognitive Processing Therapy and Relapse Prevention for individuals with PTSD and alcohol use disorder, dropout rates were 47% and 42%, respectively, with 23.7% (Relapse Prevention) and 31.7% (Cognitive Processing Therapy) discontinuing during the 12‐session active treatment phase (prior to the immediate post‐treatment assessment), and additional attrition observed at follow‐up timepoints [24]. Comparable challenges with treatment retention have also been observed in non‐trauma‐focused interventions. In a study of women with PTSD and alcohol use disorder following intimate partner violence who were randomised to Seeking Safety or Facilitated Twelve‐Step treatment, 55% of participants did not complete treatment [25]. This is concerning as greater treatment attendance is associated with greater reductions in PTSD symptoms [26].

As a result of high attrition, recent work has examined alternative delivery models of PE, including shorter sessions, massed protocols, telehealth delivery and brief primary care adaptations, suggesting that these approaches can retain treatment effectiveness while improving accessibility and reducing barriers to care [27]. Consistent with this emerging evidence, a randomised trial comparing massed PE (M‐PE; i.e., multiple sessions per week) and intensive outpatient PE reported high overall treatment engagement, with 74.4% of participants completing treatment, alongside significant reductions in PTSD symptoms and remission rates exceeding 50% at follow‐up [28]. Thus, one method for decreasing attrition rates is using M‐PE. Usually, standard PE is delivered in weekly sessions over the course of 8–15 weeks [19]; however, M‐PE may lead to increased retention and better outcomes. Recent findings [29] showed that M‐PE, delivered as three or more sessions per week, over the course of 2 weeks for a total of 10 sessions, produced significant reductions in PTSD symptoms with effect sizes comparable to or greater than standard PE, and attrition was substantially lower (14% vs. 33%), indicating that M‐PE may improve treatment completion.

### 1.3 The Implementation Gap

Despite its efficacy, PE, including M‐PE, is not widely offered in SUD care [20]. Reasons for limited availability of PE/M‐PE include: (i) concerns about client's ability to tolerate treatment and potential for relapse [30]; (ii) lack of provider training [8]; and (iii) logistical barriers (e.g., limited providers, scheduling barriers). Traditional PE delivery is often incompatible with the typical length of stay in residential or intensive treatment programs, which typically last about 30–45 days on average [31]. As a result, many clients with PTSD + SUD do not have sufficient time to complete PE before discharge. M‐PE aligns more with the intensity and frequency of comprehensive outpatient or residential programs. Research looking at PE in residential SUD treatment has also shown near‐perfect completion rates for PE in these settings [32, 33], suggesting M‐PE can address logistical barriers. Successful implementation of PE/M‐PE in SUD programs requires buy‐in from stakeholders to ensure necessary resources are available [34, 35]. However, relatively little is known about the perspectives of PE/M‐PE implementation among community SUD treatment care teams. Incorporating these voices in the evaluation of M‐PE in SUD settings, is critical for improving feasibility and sustainability [36].

### 1.4 Study Rationale and Aims

Given the strong evidence base for PE in PTSD+SUD populations and its structured, manualised protocol, coupled with emerging strong support for M‐PE, and relatively fewer randomised trials evaluating other trauma‐focused treatments such as EMDR in PTSD + SUD populations, PE was selected as the trauma‐focused intervention examined in this study. Despite growing evidence supporting trauma‐focused interventions, integrating trauma‐focused treatments into community‐based SUD programs remains challenging, although evidence demonstrating its effectiveness for individuals with PTSD + SUD. The present study explores perceptions of feasibility, acceptability and potential challenges of implementing M‐PE within a comprehensive outpatient SUD treatment program. Specifically, this study examines diverse staff perspectives, including providers, peer specialists and administrators, regarding the implementation of PE/M‐PE within a community‐based SUD program. Findings will inform future implementation strategies when integrating trauma‐focused treatments into SUD treatment settings.

## 2 Method

### 2.1 Participants and Setting

Participants were recruited to participate in a qualitative interview prior to the start of treatment delivery for Project COMET, a Hybrid—Type 1 effectiveness–implementation randomised controlled trial evaluating the delivery of M‐PE for PTSD + SUD in a comprehensive community‐based nonprofit substance use treatment organisation in the Southeastern United States. The site provides a continuum of care including detoxification, residential treatment, comprehensive outpatient services and recovery housing. Services at the site include individual and group therapy, 12‐step programming, family counselling, medication management, and recovery support services informed by motivational interviewing and cognitive behavioural therapy. Trauma‐related care within TAU consists of trauma resiliency groups focused on coping skills and symptom management modelled with input from the Seeking Safety curriculum. Clients access services through multiple pathways, including self‐referral, primary care provider referral, referrals from other agencies and court‐mandated pathways (e.g., Department of Children and Families involvement). In addition, same‐day walk‐in services are available through the Treatment Today program, which allows clients to receive an initial assessment and initiate outpatient group treatment on the same day. Recruitment occurred through distribution of study information prepared by the research team, which was disseminated to staff by site leadership electronically. Staff interested in participating contacted the research team directly using the information provided in these materials. A total of 15 staff members participated in the study, including providers (i.e., counsellor/clinicians and medical providers; *n* = 8), administrators (*n* = 2) and peer specialists (*n* = 5). See Table 1 for respondent characteristics. Providers included licensed clinical social workers, master's‐ and doctoral‐level therapists, licensed marriage and family therapists, and nurse practitioners. Administrators represented individuals in program leadership and supervisory roles responsible for overseeing clinical services, program operations and coordination of treatment delivery within the facility.

**TABLE 1:** Participants characteristics for sample.

Characteristic	Total sample (*N* = 15), *n* (%) or M (SD)
Age	
18–24 years	1 (6.7%)
25–34 years	6 (40.0%)
35–44 years	2 (13.3%)
45–54 years	2 (13.3%)
55+ years	4 (26.7%)
Sex	
Male	3 (20.0%)
Female	13 (80.0%)
Race/ethnicity	
White/European ancestry	9 (60.0%)
Black or African American	5 (33.3%)
Other	1 (6.7%)
Role	
Counsellor/clinician	5 (33.3%)
Medical provider	2 (13.3%)
Administrators	2 (13.3%)
Peer support specialist	5 (33.3%)
Other	1 (6.7%)

*Note:* Percentages reflect valid responses and are calculated relative to the total sample size (*N* = 15). Small deviations from 100% in subgroup totals reflect rounding. Age data were aggregated into broader categories to maintain participant confidentiality.

### 2.2 Study Design and Procedures

All study procedures were approved by the Rutgers University Institutional Review Board. Participation was voluntary and all participants provided informed consent prior to completing the interview. To minimise risk of coercion, recruitment and consent were conducted by Rutgers University research staff who were independent of the clinic and its program leadership. Participants were assured their leadership would not know if they participated in the study and they could decline to answer any question or withdraw at any time without penalty. Each participant was assigned a unique study ID prior to their interview and instructed to join a Zoom call using that ID. To ensure confidentiality, no site personnel were informed of who participated, and they did not have access to collected data. With participant consent, interviews were recorded using Zoom, with automated transcription and secure, cloud‐based storage. Audio files and transcripts were transferred to a secure institutional server outside the organisation for verification and analyses. A semi‐structured interview guide was developed to examine providers' understanding and experiences with trauma treatment. Participants were asked questions across 11 domains including familiarity with PE, perceptions of feasibility and barriers to implementation, and anticipated client responses. The guide was refined based on feedback from the Rutgers University Center of Alcohol and Substance Use Studies Community Advisory Board to ensure questions were clear, and relevant to the experiences of people in recovery, providers and peer providers. Staff were first asked about their prior knowledge of trauma‐focused treatments and their initial reactions to PE after being provided with a brief description of the therapy. The interview then explored perceptions and experiences with trauma treatment within SUD populations. Additional questions addressed anticipated barriers to engagement, and barriers to delivering PE or M‐PE in addiction treatment programs (e.g., resources, training and organisational support). Participants were asked to reflect on the perceived benefits of integrating trauma therapy into SUD care and their expectations for how clients might respond to PE. Finally, staff were invited to share additional reflections (see Appendix A for interview guide).

Interviews were conducted by two doctoral‐level researchers and one master's‐level research staff member affiliated with Project COMET. Interviewers received training by the Project COMET qualitative expert, which included review of the interview guide, discussion of study aims and guidance on interviewing procedures. During training, the interview team met after each practice interview to review modifications to the interview guide. After the interview guide was finalised, the team met weekly throughout data collection to resolve questions and adjust the interview guide as needed. Once all interviews were completed, the study team discussed emerging themes. Interviews lasted approximately 45 min and participants were given a $75 gift card as compensation.

### 2.3 Data Analysis

Rapid qualitative analysis framework was used to capture staff perspectives on implementing M‐PE. This method emphasises data reduction through structured summaries that distil the most relevant content from each interview [37, 38]. Structured templates derived from the interview guide were created to condense and organise the content of each transcript. To ensure consistency, seven transcripts were summarised by two team members and compared for agreement by additional research staff with prior qualitative experience. Once reliability in the summarisation process was established, each interviewer proceeded to independently complete summaries for four transcripts. The structured templates captured content related to primary interview questions and incorporated quotes to illustrate staff perspectives. Using matrix analytic techniques, members of the team organised these summaries into a data matrix displaying responses by domain and participant role (e.g., provider, administrator, peer support specialist). This approach facilitated comparison across staff categories and highlighted convergent and divergent perspectives. The matrix was then reviewed during subsequent team meetings, where interpretations were discussed and refined, resulting in a final version that captured key themes and representative quotations.

## 3 Results

We identified four key themes shared by participants: (i) Prior Knowledge and Experience; (ii) Perceptions of PE/M‐PE; (iii) Symptom Reduction and Client Impact; (iv) Barriers and Constraints. Themes and the subthemes are outlined in Table 2.

**TABLE 2:** Overview of themes.

Key themes and subthemes
1. Prior knowledge and experiences 1.1 Awareness of evidence‐based trauma treatment 1.2 Limited exposure to Prolonged Exposure (PE) and Massed PE (M‐PE) 1.3 Lived experience and peer perspectives 2. Perceptions of M‐PE 2.1 Recognised benefits of M‐PE 2.2 Concerns about timing, readiness and client stability 3. Symptom reduction and client impact 3.1 Trauma treatment beneficial for comorbid post‐traumatic stress disorder and substance use disorder 3.2 Peer specialists vs. administrators and providers' main focus when it comes to symptom reduction 4. Barriers and constraints 4.1 Logistical barriers 4.2 Client readiness barriers 4.3 Support barriers

*Note:* Study staff met to discuss subthemes that emerged from the larger four main themes. These subthemes are reflected in the subsequent analyses.

### 3.1 Theme 1—Prior Knowledge and Experiences

Staff reported limited familiarity with evidence‐based trauma therapies, with Eye Movement Desensitisation and Reprocessing (EMDR) as the most recognised and frequently mentioned approach. Some staff spoke of observing or training in EMDR as it is utilised at the site.

I know we do EMDR [at treatment site] … I feel like … that is kind of marketed as like the gold standard for trauma treatment.

Beyond EMDR, knowledge of other trauma treatments was less common. One provider referenced Seeking Safety, a present‐focused, non‐trauma‐focused intervention developed for individuals with co‐occurring PTSD and SUD [39], from their personal participation and clinical use with patients. An additional provider expressed knowledge of trauma‐focused cognitive behavioural therapy, an evidence‐based therapy addressing the psychological needs of youth who have experienced a traumatic event [40].

Seeking Safety is one that I've actually participated in. And I've used as well with the patients, which I love and I actually use it in my own personal life as well.

Direct Knowledge of PE or M‐PE was rare, with most staff only becoming aware of this treatment after receiving an offer of PE training through Project COMET. However, many staff members expressed interest in being trained in PE and recognised potential advantages of massed delivery for clients in short‐term residential care.

I requested to be trained in [PE]. So, I mean, I'm interested in it. I'm open to, you know, learning about it and doing it and applying it.

Peer specialists often described trauma therapy from their lived experience, emphasising the impact trauma treatment had in their recovery. Many peer specialists described trauma treatment as an integral part of their sobriety.

We did EMDR when I was in treatment … I realised that when I was in addiction, I was very reactive with people and it went back to the way my parents were around me. And then we worked on a lot of calming and grounding techniques.

### 3.2 Theme 2—Perceptions of M‐PE

Staff expressed generally positive attitudes towards PE, viewing it as a needed structured intervention for addressing trauma in SUD populations. Many saw value in M‐PE as a way to accelerate recovery and provide continuity, with some drawing comparisons to EMDR and other exposure‐based approaches. However, participants noted concerns about its intensity, timing, and feasibility in certain treatment contexts.

Providers emphasised that PE could be beneficial for relapse prevention and trauma recovery but stressed the importance of tailoring to client readiness and stability. Several providers expressed caution about potential harm if introduced in early stages of treatment entry or without proper support.

I feel that it will be beneficial. I would say expectations of [PE] would be that it might be a little slow … to see the benefits, but that they will eventually get there. We don't want to do more harm. You have to tailor it to the person … maybe this prolonged exposure modality could be more befitting this person opposed to EMDR.

As long as they're going to be in it for a while. I don't think it's something you want to open up and then only have two sessions with them.

I liked what I was reading about it being either multiple times a week, or at least a higher frequency, so that they have that added support.

What comes to my mind initially is like, that's kind of what is done for OCD, exposure therapy … I'm assuming it's like the similar.

Providers acknowledged logistical constraints including work schedules, transportation, and continuity of care, emphasising the importance of psychoeducation and connection‐building before starting M‐PE. Many participants, particularly administrators, believed that shorter intervals between sessions could be preferable, as this structure may reduce the time clients could become overly distressed and promote faster symptom resolution. Administrators recognised the potential benefits and risks of implementing M‐PE, emphasising a need for caution. This perspective may reflect that leaders often prefer decisions that minimise risks when designing and implementing administrative procedures, indicating that in their role they have to consider problems that may arise.

That is kind of my first take a deep breath … I guess because I recognise the significance of the impact if not done correctly. Could create more harm. That is really always my biggest concern.

Curiosity maybe create[s] that little apprehension, but I think once they really get an understanding … it would really take hold. I think it will really, really be another beneficial service.

Some comments from providers appeared to reflect misunderstandings about the structure and purpose of exposures in PE. This underscores the importance of education around the gradual, voluntary, and guided nature of exposure with clients for whom trauma‐focused therapy may be appropriate.

Some patients might think, oh wow, this sounds like a great opportunity I could work on my trauma … and then the other view might be, well, this is almost torture, making someone sit through their trauma like that. How can that be treatment?

Administrators distinguished between treatment settings, cautioning that the emotional intensity may be overwhelming in residential programs, where clients are early in sobriety, but saw outpatient programs as a better fit once stability is established.

My initial reaction was it definitely has to be patients that are stable … in inpatient, everything's raw … it might be too overwhelming. But at the outpatient level, I see it being beneficial.

We couldn't do this in detox … You wouldn't want to start something like this in detox and then they discharge and then they don't continue. That could be detrimental to the patient.

They also noted how M‐PE's accelerated format could be an advantage in real‐world practice, compressing the benefits of longer‐term treatment into a shorter timeframe which was seen as an asset for SUD settings where time in treatment is variable.

The pros would be that the program would be over in a jiffy really quick, you get the effects of a three‐month program in the span of one month.

Peer Specialists often spoke from lived experience with trauma therapy, making strong connections between trauma treatment and recovery. They highlighted how exposure‐based work can empower clients, reduce avoidance, and build long‐term resilience.

Everything I did in trauma therapy is part of why I'm not only sober right now, but why I am able to work in recovery. I think it all stems from that.

Similar to administrators, peer specialists voiced caution, noting M‐PE exposure components could feel overwhelming if clients were unprepared. However, they emphasised that in supportive contexts, the benefits outweighed the risks. Across roles, a clear divergence emerged in how participants weighed the potential risks and benefits of M‐PE. Those with lived experience, such as peer specialists, and those working directly with clients tended to view the approach as feasible and potentially transformative, whereas administrators expressed concern about possible adverse reactions or program disruptions.

It can produce a spiral, which I've experienced too. But then coming out of it, again, it's like … I got through that without disaster happening.

If someone is not responding well to it or really having a hard time with it, just to make sure there was support on the back end to help them get through the hard stuff … or maybe it's just not the right time for them to do this intense trauma work.

### 3.3 Theme 3—Symptom Reduction & Client Impact

Participants across roles believed that PE would reduce PTSD and SUD symptoms and that trauma treatment was generally essential for long‐term sobriety.

I heard this analogy and love it, is that, if you don't address the trauma … it's like having mold in the house that's just going to keep growing. You can shut the doors to the rooms, but it's just going to permeate.

I don't think long‐term recovery or sobriety is possible without trauma counselling.

Participants believed trauma treatment enabled better functioning (emotionally, socially, and physically) in SUD patients. However, peer specialists made more links between addressing trauma and improvements in self‐esteem and positive affect, including feelings of empowerment, whereas providers and administrators emphasised symptom reduction as a goal of addressing trauma in the SUD population.

What I did in treatment … my therapist condensed months of treatment into very intense sessions … it worked for me. I was ready for it. I've also learned when there's help available in a treatment centre, the people who want it will take it.

In early recovery, certain people, places, and things that I avoided like certain aisles in the grocery store certain streets, certain activities, certain music … it's associated with a very traumatic time when I was not doing so well in my addiction. I tell [clients] as time moved on, and I created new memories associated with that event, that place, or that store, I'm able to now walk freely in public.

My hope is that they are no longer victims to their trauma, that they're able to release it and grow from it and that it trickles down so it stops generational curses of not being able to talk about it or see someone about it.

These findings suggest that SUD treatment staff are aware of the relationship between SUD and PTSD and treating both simultaneously might lead to better treatment outcomes.

### 3.4 Theme 4—Barriers & Constraints

Barriers discussed by participants typically fell into three categories: logistical, client readiness, and support barriers. Logistical barriers included potential scheduling issues when clients move to outpatient treatment, including scheduling childcare, finding transportation to and from treatment, and systematic barriers like insurance coverage.

[Barriers would include] lack of experience in a treatment setting like that. Obviously, transportation just getting to those places, having to get scheduled and go you know once or twice a week … may not be an option.

A lot of our patients here don't have their own insurance or resources.

Other logistical barriers included staffing issues within comprehensive outpatient SUD programs due to high turnover rates for therapists, funding issues and enough facility space to accommodate ongoing trauma therapy. These barriers were mentioned across groups.

Having enough providers trained and that would stay long enough to see the clients through that whole three to four month process too. I know there's a lot of high turnover.

Definitely space. Definitely the appropriate Staff. I believe it's space and it's time.

Client readiness barriers centred around the well‐being of clients, their readiness to address both SUD and trauma treatment, and concerns about how clients would tolerate exposure therapy. Client readiness was mentioned more by clinicians and administrators than peer support specialists. Providers and administrators worried about the potential of overwhelming clients and triggering relapse, expressing concerns about client motivation to participate in trauma treatment. Participants noted the necessity for educational materials to combat PE misinformation and increase the likelihood that clients engage in trauma therapy.

I'm not sure if they would feel overwhelmed by the intensity, the frequency, the duration and having that balance [of both SUD and PTSD treatments].

Prior to doing this modality do more psychoeducation like coaching to try to prepare them to do it; I received a lot of kind of like wheels turning and more interest when you could show them versus just kind of talking to them.

Support barriers included lack of community support during trauma treatment. This could be support from family, friends or counsellor and peer support specialists at SUD treatment facilities. Participants were concerned that lack of support might make it difficult for clients to have help in cases where logistical barriers occur.

Lack of support you know, nobody to bring them to the appointments or nobody's supporting them to go to the appointments.

## 4 Discussion

This study aimed to gain perspectives of incorporation of PE and M‐PE into SUD treatment from staff working in SUD treatment settings. Among participants, the integration of M‐PE in this setting was generally described as a promising and needed intervention with the potential to accelerate trauma recovery among clients with SUD. Providers and administrators underscored the importance of timing and thorough psychoeducation, whereas peer specialists emphasised how exposure‐based approaches can foster empowerment and resilience.

Overall, EMDR was the most common form of trauma treatment that staff had prior knowledge and/or experience with. Familiarity with PE or M‐PE was limited among staff prior to study involvement, however, providers indicated interest in receiving training. Peer Support Specialists also expressed positive experiences with trauma treatment. This indicates potential openness among participants to the integration of PE within this setting. Findings further suggest that staff across roles generally viewed PE as a promising intervention for reducing PTSD and SUD symptoms. Providers and administrators emphasised the value of M‐PE for symptom reduction and relapse prevention, while peer support specialists emphasised its role in strengthening coping, enhancing self‐worth, and supporting sustained sobriety. Participants across roles described how M‐PE may fit the timeline and structure of residential and comprehensive outpatient SUD programs by providing greater continuity during trauma‐focused therapy compared to other forms of trauma treatment. Participants expressed potential barriers to implementation that could limit uptake, including logistical challenges (e.g., transportation, childcare, space constraints), and high staff turnover. These findings are similar to prior qualitative work showing that both staff and clients worried trauma‐focused therapy could destabilise clients early in care, triggering cravings and relapse [6]. Further, feasibility barriers, such as limited guidance on integrating PTSD care into SUD settings, posed organisational challenges equiring system‐level solutions like training, protocols, space, and safety measures [30]. However, there is strong evidence indicating that these concerns are largely unfounded. Meta‐analytic findings show that trauma‐focused treatments can be delivered safely to individuals with PTSD+SUD and are associated with significant improvements in PTSD symptoms without worsening substance use [12] and often improve substance use outcomes [17, 18]. Consistent with this evidence, the American Psychological Association's clinical practice guidelines state that PTSD treatment should not be delayed because of ongoing substance use and identifies integrated trauma‐focused treatments as first‐line approaches for individuals with PTSD+SUD [40]. As part of the larger Project COMET study, the limited familiarity with PE identified among staff and the presence of some misconceptions about exposure‐based treatments prompted the study team to develop educational materials describing the M‐PE intervention and study procedures. Brochures summarising the treatment approach were distributed to treatment staff and made available to potential participants to support psychoeducation. These materials also included publicly available resources from the VA National Center for PTSD to provide additional information about PE therapy [41].

### 4.1 Strengths and Limitations

The present study provides insight into the implementation of M‐PE within a community SUD treatment setting. This is one of the first qualitative studies examining staff perceptions of M‐PE within this setting. The present study is unique in providing a nuanced understanding of perspectives across distinct roles (providers, peer specialists, administrators), capturing how role‐specific responsibilities, training backgrounds, and lived experiences shape attitudes towards trauma interventions such as M‐PE. It may be among the first studies to explicitly incorporate peer support specialists' perspectives of trauma treatment among those diagnosed with SUD, which is important given recent work showing that peers provide critical support in building trust, modelling recovery, and bridging communication gaps between clients and providers [42].

Though the present study had many strengths, several limitations should be acknowledged. The sample was drawn from a single SUD treatment facility in a Southeastern state, which may not be generalisable to other staff perceptions in treatment facilities in other regions. Although inclusion of multiple staff roles enhances ecological validity, we did not gather client perceptions in the present study, which may also impact implementation. Researchers' own biases may have influenced the interpretations of the present findings. To limit research bias, the team used peer debriefing, a common process in qualitative research, with experts to minimise risk of biases and ensure quality of interpretations [43].

### 4.2 Implications for Implementation

The findings from this study support implementing trauma‐focused interventions in residential and comprehensive outpatient SUD programs after navigating potential barriers. Although evidence‐based trauma interventions are recommended for individuals with PTSD + SUD, their availability in SUD programs remains limited. The perspectives identified in this study suggest that implementation challenges may stem less from resistance to trauma treatment itself and more from logistical constraints, organisational resources, and uncertainty about client readiness. Effective implementation plans should take into consideration the time and resource constraints that clients may have. Having flexible options, such as offering remote sessions, could offset transportation barriers and enable sustainability and accessibility across clients. Addressing real‐world barriers and acquiring organisation support may be key factors in implementing PE/M‐PE in SUD settings.

Findings from the current study highlight several organisational supports that may facilitate implementation, including leadership buy‐in and flexibility in treatment scheduling (remote and in‐person options). Organisational resources and funding for more time‐intensive treatments and staff training may be needed to support implementation. Future research is needed to determine whether they improve implementation of M‐PE.

Alongside organisational supports, changing staff attitudes and misconceptions about clients' well‐being and distress tolerance within trauma‐focused treatment is important for the successful implementation of M‐PE in a community‐based SUD program. Administrators highlighted potential systematic fallout if problems arose from trauma treatment (a more risk‐averse perspective), while peer support specialists were more open to the implementation of PE/M‐PE based on their personal experiences with trauma treatment. As such, seeking perspectives from diverse stakeholder roles may be beneficial when making decisions about treatment implementation. Incorporating procedures to assess and support clients in learning the benefits of this approach may also enhance interest in pursuing treatment. This includes potentially creating screening tools similar to the Cognitive Behavioral Therapy Readiness assessment tool [44] that can be used to assess client readiness and prevent premature enrolment into trauma treatment. It may also be beneficial to utilise peer support specialists to educate clients with SUD about trauma treatment to encourage engagement. Their lived experience might increase trust between clients and providers, while also providing support to clients during treatment [45, 46]. This may increase retention and prevent client dropout.

### 4.3 Future Directions

The present study provided valuable insight into current staff perceptions towards implementing trauma‐focused therapy like M‐PE into comprehensive outpatient SUD facilities; however, these attitudes were gathered before M‐PE treatment was initiated by Project COMET. A follow‐up study of this same design will be conducted after M‐PE is implemented within the larger study. This will allow comparisons of how perceptions might change as a result of implementing M‐PE.

Client perspectives on M‐PE post‐treatment will also be collected to gain insight from those participating in the treatment. This will help determine whether clients have the same concerns as staff, or if they are strictly staff attitudes. Participants noted the importance of changing staff opinions around trauma treatment for successful implementation. Future studies should examine strategies for best practices to change prior perceptions (e.g., concerns that clients cannot manage simultaneous PTSD + SUD intervention), as these views may impact buy‐in from staff and ability to deliver treatment in an unbiased way. Although not the goal of the study, qualitative interviews provided information that was integrated into the larger study to increase study feasibility (e.g., creation of brochures with information about PE/M‐PE for distribution to clients).

Future work should consider the use of Sequential Multiple Assignment Randomised Trial (SMART) designs to increase implementation [47], as SMART allows for the ability for study staff to make modifications to study design based on clients' engagement and response to treatment as well as organisational needs (e.g., space, provider training). It may also be beneficial to compare client perspectives on different treatment modalities (e.g., PE/M‐PE vs. cognitive processing therapy v pharmacological interventions). Future studies should also assess solutions for logistical barriers such as web‐based PE/M‐PE, developing a train the trainer model, or using Written Exposure Therapy or group‐based interventions to help mitigate concerns about limited space and provider time.

## 5 Conclusion

This study examined staff perspective on feasibility and potential barriers to implementing trauma treatment, specifically M‐PE, within a comprehensive outpatient SUD treatment program. Overall, attitudes towards PE were positive, with staff recognising the importance of treating PTSD and SUD in comorbid populations while also noting concerns regarding client readiness. In addition to readiness, future research should also consider client interest in engaging in trauma‐focused treatment, as understanding whether clients want to address trauma during SUD treatment may further inform implementation efforts. Other barriers listed were logistical and organisational support. Peer support specialists emphasised potential impacts on client self‐esteem and empowerment, whereas providers and administrators focused on symptom reduction. Differences in perspectives between the peer specialists and other providers highlight the importance of integrating peer‐informed approaches when adapting trauma treatments like PE/M‐PE for SUD settings, as peer support specialists' unique experiences may provide valuable insights. Together, these findings suggest that staff see PE/M‐PE as both feasible and valuable, though staff expressed that successful implementation requires attention to staff and client level factors, resource constraints, and support systems to mitigate risks.

## Author Contributions

Each author certifies that their contribution to this work meets the standards of the International Committee of Medical Journal Editors.

## Funding

This work was supported by the National Institute of Mental Health (R01MH132720‐02).

## Conflicts of Interest

The authors declare no conflicts of interest.

## Supporting information


**Appendix A.** Interview summary.

## Data Availability

The data that support the findings of this study are available from the corresponding author upon reasonable request.
