# Supplementary material for: Implementing Prolonged Exposure Therapy in a Community Substance Use Treatment Program: A Qualitative Study
Source: Drug Alcohol Rev. 2026 Aug 26;45(6):e70239. doi: 10.1111/dar.70239 (PMC13518069; doi:10.1111/dar.70239)
Supplement: Supplementary file 1 [file DAR-45-0-s001.docx]

**Appendix A**

**Interview Summary**

**Prepared by:**

**Participant ID:**

**Data Source:**

**Participant Category: Counselor/Clinician	Medical Provider	Director/Manager	Peer Specialist**

**Participant had experience with PE/ knew about PE before interview: Yes	No**

**Prior knowledge of trauma focused treatment for PTSD (thoughts when participants hear about this type of treatment):**

**Reactions to Prolonged Exposure Therapy for PTSD (based on prior knowledge/brief description of what PE is):**

**Participant’s perception of/ experiences with trauma treatment (including intensive treatment) in SUD populations:**

**Personal/Cultural beliefs that informed perceptions of seeking help for trauma:**

**Perceptions of barriers for participant engagement in PE/Intensive PE:**

**Perceptions of barriers for delivery of trauma focused care(PE and/or Intensive PE) in an addiction setting (i.e., resources needed for the implementation/delivery and patient engagement of intensive PE):**

**Perceived Benefits of trauma focused care(PE and/or Intensive PE) in an addiction setting:**

**Expectations of how clients might respond to PE:**

**Additional Reflections:**

**Other Important / Unexpected Findings:**

**Good Quotes:**
